# Open-Source Intelligence for Detection of Radiological Events and Syndromes Following the Invasion of Ukraine in 2022: Observational Study

**DOI:** 10.2196/39895

**Published:** 2023-06-28

**Authors:** Haley Stone, David Heslop, Samsung Lim, Ines Sarmiento, Mohana Kunasekaran, C Raina MacIntyre

**Affiliations:** 1 Biosecurity Program, The Kirby Institute, Faculty of Medicine University of New South Wales Kensington Australia; 2 School of Population Health, Faculty of Medicine University of New South Wales Sydney Australia; 3 School of Civil & Environmental Engineering University of New South Wales Sydney Australia; 4 College of Public Service & Community Solutions Arizona State University Tempe, AZ United States

**Keywords:** artificial intelligence, contamination, data source, early warning, emergency response, environmental health, open source, open-source intelligence, OSINT, power plant, public health, radiation, radiobiological events, radiological, sensor, Ukraine

## Abstract

**Background:**

On February 25, 2022, Russian forces took control of the Chernobyl power plant after continuous fighting within the Chernobyl exclusion zone. Continual events occurred in the month of March, which raised the risk of potential contamination of previously uncontaminated areas and the potential for impacts on human and environmental health. The disruption of war has caused interruptions to normal preventive activities, and radiation monitoring sensors have been nonfunctional. Open-source intelligence can be informative when formal reporting and data are unavailable.

**Objective:**

This paper aimed to demonstrate the value of open-source intelligence in Ukraine to identify signals of potential radiological events of health significance during the Ukrainian conflict.

**Methods:**

Data were collected from search terminology for radiobiological events and acute radiation syndrome detection between February 1 and March 20, 2022, using 2 open-source intelligence (OSINT) systems, EPIWATCH and Epitweetr.

**Results:**

Both EPIWATCH and Epitweetr identified signals of potential radiobiological events throughout Ukraine, particularly on March 4 in Kyiv, Bucha, and Chernobyl.

**Conclusions:**

Open-source data can provide valuable intelligence and early warning about potential radiation hazards in conditions of war, where formal reporting and mitigation may be lacking, to enable timely emergency and public health responses.

## Introduction

On February 24, 2022, the Russian invasion of Ukraine began. On the first day of the invasion, battles between Russian and Ukrainian forces occurred in the vicinity of the Chernobyl power plant [1,2]. Following the invasion of Chernobyl on February 25, 2022, the Ukrainian government reported increased levels of radiation in the air [3]. Unverified reports at the time raised concerns of increased radiation levels in the area, potentially due to the disruption of the soil in highly contaminated areas around the power plant due to the fighting and military vehicles moving over the exclusion zone [1,2]. Armed conflict continued throughout March 2022, with intense fighting in Slavutych, a town nearby that houses workers at the power plant. On March 22, 2022, forest fires broke out within the Chernobyl exclusion zone with the potential for generation of contaminated smoke [4]. On March 31, there were reports of confirmed radiation exposure of Russian soldiers, most likely due to soldiers digging trenches in the soil within the Red Forest area to the west of the nuclear power plant [5]. This highlighted the possibility of contamination of previously uncontaminated areas and the potential for subsequent radiological impacts on human and environmental health.

Epidemic open-source intelligence (OSINT) systems provide new approaches to public health surveillance and are increasingly used for epidemic early warning [6]. Early warning OSINT systems can complement and improve the performance of formal surveillance systems by enabling early detection of serious events or fill a gap when routine surveillance systems fail or are absent. Indicator-based surveillance systems largely require clinicians to link cardinal clinical features of specific diseases with key historical, geographic, and social data, thus recognizing the potential occurrence of disease either in an individual or in populations. This process can easily be undermined by a lack of clinical experience, biological variability of presentations in populations, and most importantly, a delay in the recognition of potential disease due to the time it takes for cardinal features to manifest in patients. By contrast, OSINT systems can provide earlier warning through the analysis of large volumes of unstructured digital data and communications. Such data do not rely on clinical experience or acumen, official health system reporting, or the results of laboratory testing. Through the use of specialized processes and algorithms, early warning of potential outbreaks of diseases in populations can be flagged from unstructured sources such as new articles and social media [7]. EPIWATCH and Epitweetr are examples of such systems [6,8]. While OSINT lacks verification, an early warning can be followed by a formal investigation to verify a signal.

Early warning for radiation effects is time critical, as exposures may result in severe outcomes and affect large populations. Following radiation and radioisotope exposure, acute radiation syndrome (ARS) can manifest as early as hours after exposure, and certain therapies require immediate delivery. During the Ukraine conflict, public health surveillance and health protection programs relevant to radiological exposures have been limited or completely ceased. Therefore, in the context of conflict and degraded public health systems, the use of OSINT to rapidly identify locations where a radiological event may have occurred is important and enables the most efficient and timely allocation of limited health resources to limit the spread and impact of contamination. A key distinction in the Ukrainian conflict, as compared broadly to other conflicts, has been the widespread and continued access to high quality open-source data communications, including social media and news sources, across the broad Ukrainian geography and continued penetration of access within the Ukrainian population. Unstructured data from local news reports, social media, and various open-source channels from the Ukrainian population and occupying forces can be used by systems such as EPIWATCH and Epitweetr to detect signals for health-related events of importance.

This study aims to demonstrate the value of OSINT in Ukraine to identify signals of potential radiological events of health significance during the Ukrainian conflict.

## Methods

### Data Collection

To determine the potential detection of radiobiological events, data were analyzed between the timeframe of February 1 and March 20, 2022, using both EPIWATCH and Epitweetr systems. EPIWATCH is an artificial intelligence (AI)-driven system that uses both curated information, such as governmental reports, and broader web searches to generate automated early warnings for epidemics worldwide [9-12]. Outbreak signals in EPIWATCH are obtained from reports collected in real time using prespecified search terms applied to open-source data. These can be monitored for deviations from baseline or unusual, newly emerging diseases. The system contains 52 translated languages, together with geographic information system capability. In addition to 2 AI subsystems (natural language processing [NLP] and a prioritization algorithm), the information collected is curated by epidemic analysts. Epitweetr is an R-based open-source data surveillance tool. Epitweetr’s data are routinely collected. In order to monitor trends in tweets’ geolocation, time, and topic using the Twitter Standard Search API, data are collected by sending queries to the predetermined list of topics and associated keywords. The default topics list consists of 71 unique topics but can be customized to the user’s choice [8,13]. EPIWATCH, at the time of this study, did not query Twitter. Epitweetr was used to enhance the data set to include social media coverage, as social media is more likely to pick up early signals for acute radiation syndrome. However, as social media is more vulnerable to manipulation, both systems are needed to validate potential detections or events.

These systems were originally created to detect infectious disease outbreaks but can be rapidly adapted for the detection of radiobiological events. A series of search terms were created by a domain expert on radiation (DH) that were indicative of potential radiobiological events (acute exposure to radioisotopes, contamination by radioisotopes, ARS, and related medical symptoms and signs). The terms were translated into Ukrainian and Russian. The search terms are listed in Table 1 and their definitions are in Multimedia Appendix 1. In addition, since users often do not disclose direct illness on social media but rather discuss symptoms, we individualized each symptom and added variations for analysis for radiation poisoning. Symptomology terms for acute radiation poisoning were also investigated and are described in Table 2. Reports collected from EPIWATCH were obtained through a manual search within the system; they did not undergo machine learning classification and were gathered solely through noncurated broader web searches using Google Alerts (Table 3). Data collected through Epitweetr queried the terms added through the Epitweetr Shiny app interface using Twitter APIs 1 and 2. The tweets gathered from the queries are then aggregated and geolocated, and an Early Aberration Reporting System (EARS) signal detection algorithm is applied. Each individual tweet is counted as a report and is visualized in the results (Table 3) [8,13]. Data collection occurred after the search timeframe for potential radiobiological events, from March 20 to April 12, 2022. Both systems used the same search terms to investigate the potential for radiobiological events and acute radiation poisoning, which are justified and explained in Multimedia Appendix 1 and Tables 3 and 1. Specific terms related to features of acute radiation exposure (eg, radiation types, the Cherenkov effect, radioisotopes, and initial medical impacts) and terms relating to the short-term effects and immediate medical management of exposure were used.

**Table 1:** Terms used in the search for radiobiological events in Ukraine by subtopics: event-based terms (n=14), radiological substance–based terms (n=14), medical terms (n=13), and radiation preparedness terms (n=8).

English			Ukrainian		Russian
**Event-based terms**					
	Radiation	Радіація		Радиация	
	Radiological	Радіологічний		Радиологический	
	Reactor	реактор		Реактор	
	Alpha radiation	Альфа-випромінювання		Альфа-излучение	
	Beta radiation	Бета-випромінювання		Бета-излучение	
	Gamma radiation	Гамма-випромінювання		Гамма-излучение	
	Isotope	Ізотоп		Изотоп	
	Geiger	Гігер		Гигер	
	Curie	Кюрі		Кюри	
	Becquerel	Бекерель		Беккерель	
	Sievert	Зіверт		Зиверт	
	REM^a^	REM		REM	
	RAD^b^	RAD		RAD	
	Cherenkov	Черенков		Черенков	
**Radiological substance–based terms**					
	Iodine	йод		Йод	
	I-131	І-131		І-131	
	Cesium	цезій		Цезий	
	Cs-137	Cs-137		Cs-137	
	Cs-134	Cs-134		Cs-134	
	Plutonium	плутоній		Плутоний	
	Strontium	стронцій		Стронций	
	Sr-90	Sr-90		Sr-90	
	Americium	америцій		Америций	
	Am-241	Ам-241		Ам-241	
	Uranium	уран		Уран	
	Nuclear fuel	Ядерне паливо		Ядерное топливо	
	Nuclear waste	Ядерні відходи		Ядерные отходы	
	Graphite	Графіт		Графит	
**Medical terms**					
	Beta burn	Бета-запис / Бета опік		Бета ожог	
	Desquamation	Десквамація		Десквамация (latin) / Шелушение	
	Hair loss	Втрата волосся		Выпадение волос	
	Mucositis	Мукозит		Мукозит	
	Gastrointestinal syndrome	Шлунково-кишковий синдром		Желудочно-кишечный синдром	
	Cardiovascular syndrome	Серцево-судинний синдром		Сердечно-сосудистый синдром	
	Neurological syndrome	Неврологічний синдром		Неврологический синдром	
	Melena	Мелена		Мелена	
	Vomiting	Блювота		Рвота	
	Lymphopaenia	Лімфопенія		Лимфопения	
	Bone marrow suppression	Пригнічення кісткового мозку		Подавление костного мозга	
	Bone marrow transplant	Пересадка кісткового мозку		Пересадка костного мозга	
	Sepsis	Сепсис		Сепсис	
**Radiation preparedness terms**					
	Potassium Iodide	Калій йодид / Йодистий Калій		Йодистый калий	
	Heavy Metal Chelation	Хелатування важких металів		Хелотирование тяжелых металлов	
	Calcium DTPA	Кальцій DTPA		Кальцый DTPA	
	Zinc DTPA	Цинк DTPA		Цынк DTPA	
	Decontamination	Дезактивація/ знезараження		Деконтаминация / Обеззараживание	
	Prussian Blue	Прусський блакитний/ берлінска блакитність		Берлинская лазурь	
	Granulocyte Monocyte Colony Stimulating Factor	Фактор, що стимулює колонію гранулоцитів моноцитів		Фактор стимулирующий колонию гранулоцитов моноцитовю / Гранулоциты Моноциты Колониестимулирующий фактор	
	Granulocyte Colony Stimulating Factor	Фактор, стимулюючий колонію гранулоцитів		Фактор стимулирующий колонию гранулоцитов / Гранулоцитарный колониестимулирующий фактор	

^a^RAD: radiation absorbed dose.

^b^REM: roentgen equivalent man.

**Table 2:** Syndromic terms and variants of each term used to search for acute radiation poisoning in Ukraine [14].

Syndromic terms	English	Ukrainian	Russian
Radiation	radiation OR RAD OR radiated OR glowing	випромінювання АБО RAD АБО випромінюваний АБО світиться	излучение ИЛИ RAD ИЛИ излучаемое ИЛИ светящееся
Nausea	nausea OR nauseated	нудота АБО нудота	тошнота ИЛИ тошнота
Vomiting	vomiting OR vomit OR throwup OR puke	блювота АБО блювота АБО блювота АБО блювота	рвота ИЛИ рвота ИЛИ рвота ИЛИ рвота
Headaches	headaches OR headache OR migraine	головні болі АБО головний біль АБО мігрень	головные боли ИЛИ головная боль ИЛИ мигрень
Fatigue	fatigue OR drowsy OR disoriented	втома АБО сонливість АБО дезорієнтація	усталость ИЛИ сонливость ИЛИ дезориентированность
Fever	fever OR feverish OR temperature OR shivering	гарячка АБО лихоманка АБО температура АБО тремтіння	лихорадка ИЛИ лихорадка ИЛИ температура ИЛИ озноб
Rash and fever	skin-reddening OR rash	почервоніння шкіри АБО висип	покраснение кожи ИЛИ сыпь

**Table 3:** Data output for Epitweetr and EPIWATCH.

System	Output data type	Data analysis before output
Epitweetr [8,13]	Aggregated tweets by search term	Twitter results for individual terms are initially geolocated. The tweets are then aggregated on terms and geolocation. Finally, the Early Aberration Reporting System algorithm is applied to identify if a signal was detected by qualitatively comparing baseline activity to aberrations (2 standard deviations) from the baseline.
EPIWATCH	Web results by search term	Manual search through the EPIWATCH system by term is performed by a human analyst. Each individual web result is deemed as a report. Aggregate by term is performed manually. Data generated are reviewed to identify if a signal was detected by qualitatively comparing the baseline activity to aberrations (2 standard deviations) from the baseline.

### Data Analysis

A comprehensive line list was created for both EPIWATCH and Epitweetr. Analysis was completed separately on potential radiological events and acute radiation detection analysis. Data were sorted using MATLAB by date, subtopic, language, and, for Epitweetr, subnational geolocation. Data from Epitweetr were only used if the tweet’s geolocation was within Ukraine. Analysis and reporting for this study followed STROBE (Strengthening the Reporting of Observational Studies) guidelines for epidemiological studies [15]. For EPIWATCH’s signal reports, for both radiobiological event analysis and acute radiation detection analysis, a mean daily signal count (*σ*) was established for each subtopic using Stata/IC (Stata Corp). The total daily signal was adjusted for this factor. The signal curve was constructed using the date of the signal and the total adjusted daily signal. The plots were analyzed on the date of peak signal and compared to key dates and events around Ukraine. We used the data to identify if a signal was detected by qualitatively comparing baseline activity over time to aberrations (2 standard deviations) from baseline and in relation to key events around the war. Analysis of Epitweetr included searches for the individual terms in order to identify increased signals within the given time period. In addition, geolocation of the total signal amount and subtopic were performed using descriptive statistics and plotted using ArcGIS Pro (Esri).

### Ethical Considerations

This study only contains open-source data which are publicly available. No individual or identifying data about patients or people were collected. In addition, all the data presented are in aggregate form and have been deidentified before data analyses were completed.

## Results

### Potential Radiobiological Event Detection

**Figure 1:**
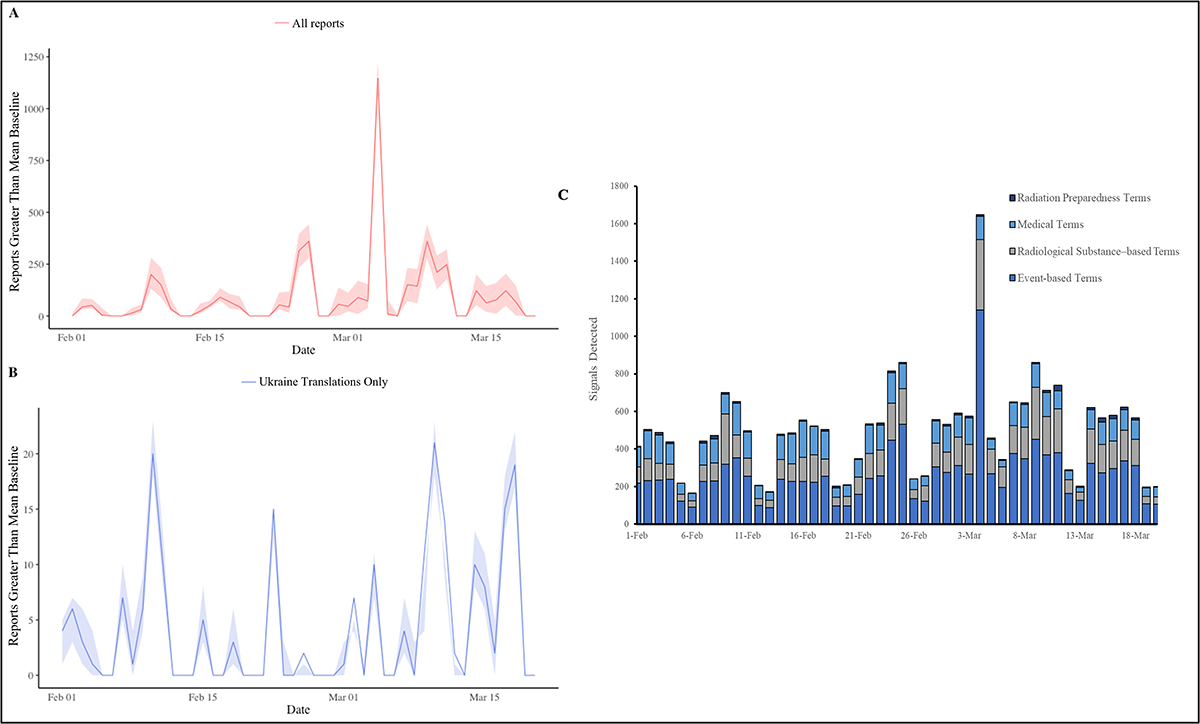
EPIWATCH’s adjusted daily signal detection for all languages (A) and Ukrainian (B) were examined in addition to a time series by subtopic (C).

Both systems detected potential radiological events from February 1 to March 20, 2022. Terms used to mine these open-source news were separated into 4 subgroups: event-based surveillance, radiological substance, radiological preparedness, and medical based terms. EPIWATCH overall detected 24,071 reports with a mean of 502 reports per day (*σ*=36.6; 95% CI 427.8-575.2) with an adjusted peak on March 4 (n=1147) using both English and Ukrainian translations. Of the reports, 5.6% (n=1348) were Ukrainian. Adjusted daily reports for both English and Ukrainian translations found 5 distinct peaks on February 24 and 28 and March 4, 9, and 17 (Figure 1A). Likewise for Ukrainian-only translations, 5 peaks were observed on February 10 and 23 and March 4, 10, and 18 (Figure 1B).

**Figure 2:**
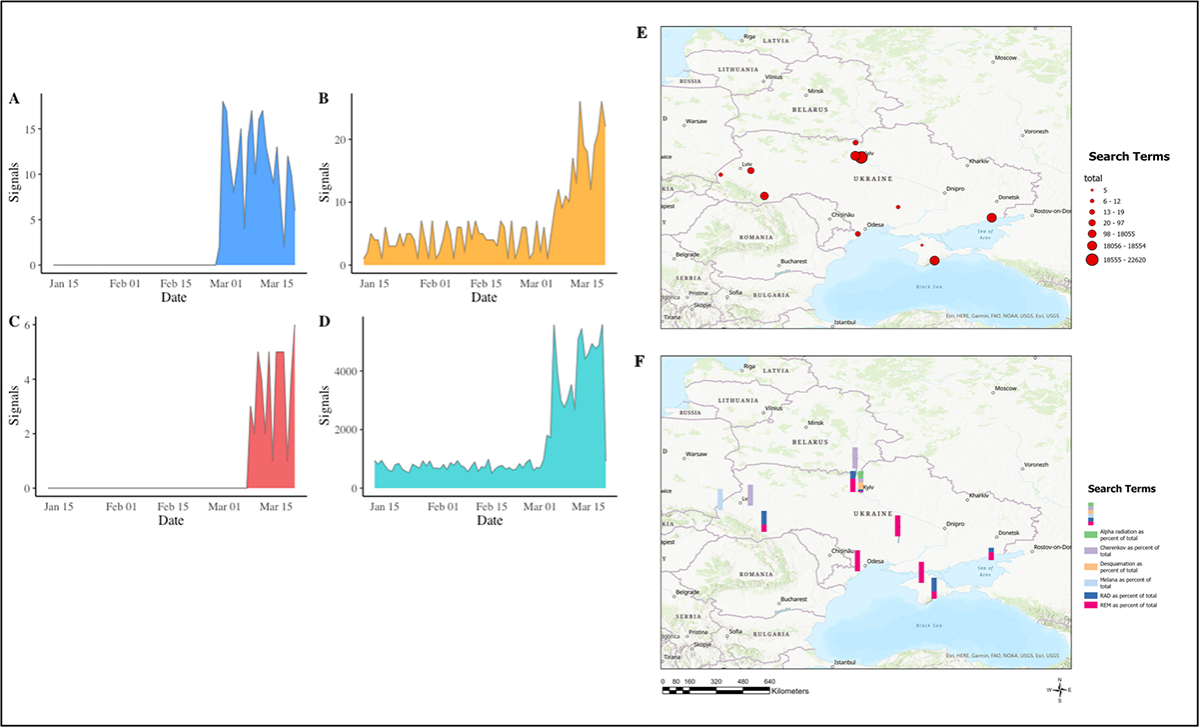
Epitweetr’s signal (tweets) detection for (A) Cherenkov, (B) roentgen equivalent man (REM), (C) alpha radiation, and (D) radiation absorbed dose (RAD). Event and radiological terms by (E) region (n=96,094) and (F) individual term.

Event-based surveillance terms, described in Multimedia Appendix 1, were detected with a daily mean of 265 (*σ*=23.9; 95% CI 216.7- 312.8) reports and had 2 peaks from February 24 and March 4 (Figure 2C). For radiological substance–based terms, EPIWATCH detected a mean of 123 reports per day (*σ*=10.1; 95% CI 102.2- 142.9) and had a peak on March 4 (n=376). For medical-based terms, EPIWATCH detected a mean of 106 reports per day (*σ*=6.1; 95% CI 94.0-118.3) and had a peak on February 24 (n=162). Lastly, for radiation preparedness terms, EPIWATCH detected a mean of 8 reports per day and had a peak of reports on March 11 (n=29) (Figure 1B). Using exclusively the Ukrainian translations, EPIWATCH detected a mean of 27 reports per day (*σ*=1.3; 95% CI 24.0-29.4) and a peak observed on March 18 (n=46) for event-based surveillance terms. For radiological substance-based terms, EPIWATCH detected a mean of a report per day (*σ*=0.3; 95% CI 0.6-1.9) and peaks on March 2, 9, and 11 for radiological substance-based terminology. For medical-based terms, EPIWATCH detected a mean of less than a report per day with single reports found on March 3 and 17. Lastly, for radiation preparedness, no reports were detected with Ukrainian translations.

For Epitweetr, a total of 4 different search terms were identified to have distinct peaks during the invasion in Ukraine: Cherenkov radiation, which was first reported on February 28 and peaked on February 28 (Figure 2A); REM, which rose from the baseline average of 4 signals a day on March 3 and peaked twice on March 13 and 19 (Figure 2B); alpha radiation, which was first reported on March 8 and peaked on March 20 (Figure 2C); and RAD, which rose from the baseline average of 743 signals per day on March 2 and had 3 peaks on March 4, 12, and 20 (Figure 2D).

The 3 highest regions within Ukraine for the radiological terms were within Kyiv (22,620/96,094, 23.5%), Semidvor’e (18,405/96,094, 19.2%), and Mariupol (18,301/96094, 19%) for Epitweetr (Figure 2E). Additionally, a total of 2 terms reported signals within the Chernobyl area: *Cherenkov* (n=12) and *RAD* (n=4) (Figure 2F).

### Potential Acute Radiation Syndrome Detection

EPIWATCH detected 51,248 reports of symptoms related to radiation poisoning throughout the period between February 24 and March 20, 2022 with a mean of 2050 reports per day (*σ*=131.7; 95 CI 1778.1-2321.8) and an overall peak on February 28 (n=2898). Radiation reports had a mean of 354 (*σ*=32.6; 95% CI 286.5-421.3) and a noticeable peak on March 4 (n=940), which consisted of 54.4% of all reports on 1 day. Nausea reports had a mean of 38 (*σ*=2.8; 95% CI 31.8-43.2) and a peak on March 7 (n=66). Vomiting reports had a mean of 44 (*σ*=2.5; 95% CI 39.3-49.4) and a peak on March 1 (n=65). Headache reports had a mean of 147 (*σ*=10.1; 95% CI 125.8-167.4) and a peak on March 2 (n=231). Fatigue reports had a mean of 153 (*σ*=9.8; 95% CI 133.2-173.6) and a peak on February 26 (n=210). Fever reports had a mean of 624 (*σ*=43.2; 95% CI 534.4-712.84) and a peak on February 28 (n=1001). Skin-reddening reports had a mean of 44 (*σ*=2.8; 95% CI 37.6-49.3) and a peak on March 1 (n=78). Rash and fever reports had a mean of 5 (*σ*=0.7; 95% CI 3.1-6.0) and a noticeable peak on March 1 (n=14).

**Figure 3:**
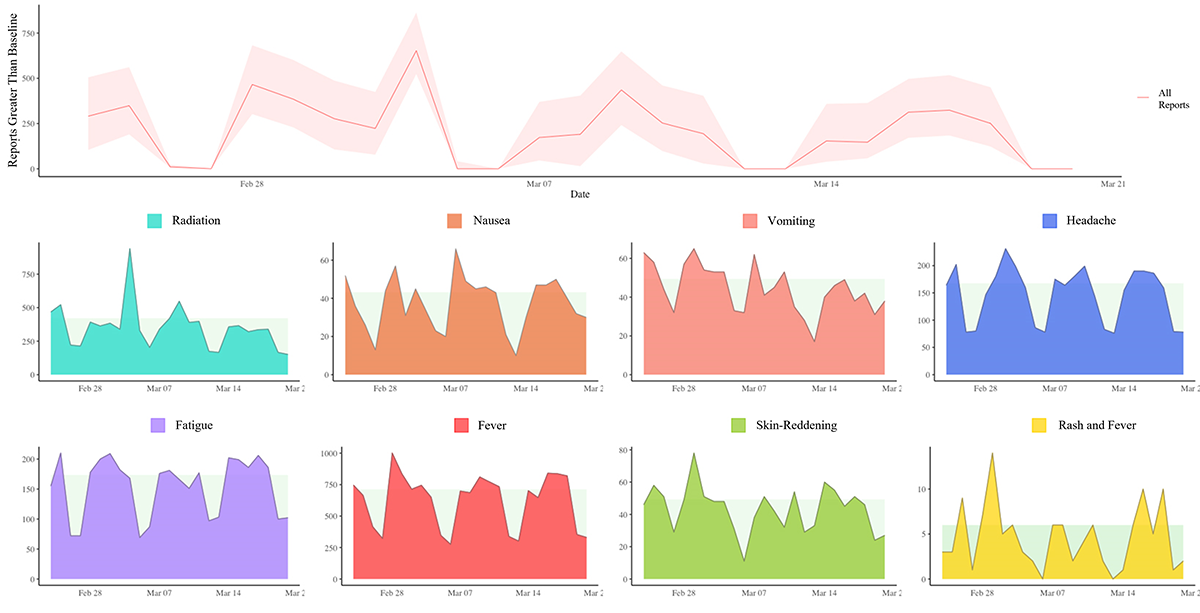
EPIWATCH reports on syndromes for acute radiation poisoning by detecting reports above the baseline daily mean and by individual syndromes between February 24 and March 20, 2022, (n=51,248).

**Figure 4:**
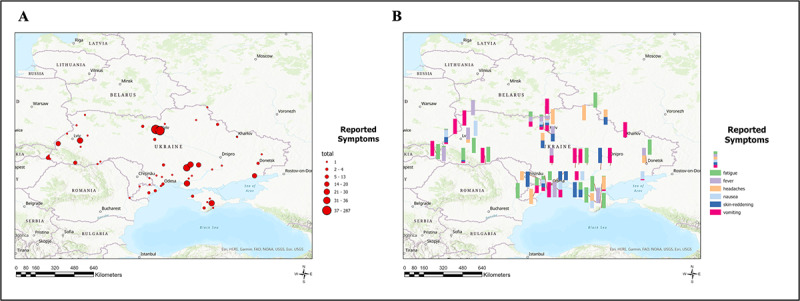
Epitweetr’s signals reported by region for both total reports per region (A) and by individual symptom per region (B) (N=757).

A total of 757 signals were detected with the symptomology related to radiation poisoning from Epitweetr. The 2 regions with the most signals detected were within Bucha (n=287) and Kyiv (n=196) at the time of the search (Figure 3A). Of the 757 detected signals for the symptoms related to radiation poisoning, 27.6% (n=209) signals were for vomiting, 24% (n=182) were for fever, 15.6% (n=118) were for nausea, 15.3% (n=116) were for skin reddening, 10.3% (n=78) were for fatigue, and 7.1% (n=54) were for headaches (Figure 4B).

## Discussion

We have shown that under conditions of war, when routine reporting and monitoring may be disrupted or absent, open-source intelligence from news reports or social media can be used for early warning of potential radiation events. While signals were detected on February 24 with the beginning of the invasion of the Chernobyl plant, both systems detected further signals in March, which could be linked to the rise in radiobiological events, such as radiation exposure during the armed conflict within the exclusion zone. The Russians occupied the Chernobyl plant from February 24 to March 31, 2022 with acute radiation syndrome reported in Russian soldiers on March 31 and one death reported [5,16]. Potential exposure could have been throughout the occupation of Chernobyl and surrounding areas by the Russian soldiers. Additionally, there was global concern about the disruption of Chernobyl and other nuclear sites during the invasion. Geolocation analysis of radiobiological events for Epitweetr found 2 terms within the Chernobyl region: *RAD* and *Cherenkov*. For acute radiation poisoning syndromic analysis, *vomiting* and *headaches* were identified within regions surrounding the Chernobyl exclusion zone in the month of March. Clustering of signals in our syndromic analysis for radiation sickness appeared in or around Kyiv. The results from this study show the usefulness of immediate, timely information, particularly in a war zone where access for investigations might be minimal. This information, obtained rapidly, can complement the formal intelligence systems already in place.

OSINT systems have already been used in Ukraine to aid in the detection of potential war crimes and military movements [17]. We showed that OSINT can detect potential radiation events and can be used in real time for early warning. While not a replacement for validated data, such as radiation measurements, open-source data can provide early intelligence when formal reporting is absent and can provide a trigger for an early investigation or emergency response.

A potential limitation to using open-source data is the possibility of manipulation or interference by third parties through the injection of tweets or news sources to boost sentiment. This can be mitigated through multi-source data fusion, triangulation of data, and correlation within and between NLP and machine learning (ML) identified data and other sources of data. In this study, the use of news-based OSINT allowed the validation of Twitter-based OSINT. EPIWATCH, an AI system, applies a model using contemporary NLP and named entity recognition (NER) algorithms in order to detect unusual spikes or signals in particular topics. In addition to system filtration, human moderation is implemented to verify the authenticity of reports. Epitweetr, unlike EPIWATCH, does not individually filter tweets but instead uses a modified EARS, which is a well-established model developed by the US Centers for Disease Control as a baseline for signal detection. OSINT can result in lexical bias that can lead to overreporting of signals and cause issues establishing signal validity. The bias can be allayed by using specific terminology to decrease irrelevant outside noise. This mitigation was confirmed by the detection of distinct spikes in specific terminology not used in regular vernacular.

A further limitation of this study is the dependence on the quality of the data inputs. The data obtained from EPIWATCH could have delayed reporting time or contain biases. There are also language biases, with a predominant amount of news reports being in English for EPIWATCH, despite searching in Ukrainian, which could indicate events outside the scope of the Ukrainian invasion. However, we did perform an analysis on the total reports from EPIWATCH in addition to solely reports in Ukrainian to detect varying signals, if any, from the 2 languages. Additionally, searching in Ukrainian only began in February 2022, whereas searching in Russian was part of EPIWATCH since 2019. For Epitweetr, the tweets are aggregated and rely on built-in signal detection algorithms to distinguish actual events from “white noise.” In addition, the symptoms of radiation poisoning can be indicative of other diseases rather than radiation poisoning. We, however, attempted to mitigate by clustering and geolocating symptoms, in which all symptoms in our syndromic analysis appeared in or around Kyiv. Lastly, the signal detected using open-source syndromic analysis may not reflect radiation exposure and may be a false positive. However, the purpose of OSINT is to monitor the baseline, detect early warning signals above the baseline, and then formally investigate for confirmation.

Using OSINT systems such as EPIWATCH and Epitweetr, signal detection from war zones can be used in the absence of formal detection methods to help rapidly discover and control public health risks. Several studies have identified social media, particularly Twitter, that can be used to identify particular syndromes [18-20]. The value of these open-source data systems, like Epitweetr and EPIWATCH, is the rapid detection of outbreaks and public health events when surveillance systems are not as robust or have been weakened, such as with the invasion of Ukraine [21,22]. An estimated 50% of the stakeholders in epidemic response report lacking access to timely surveillance data, yet 90% do not use available open-source systems, highlighting the potential to improve the use of OSINT [9].

Both systems identified potential radiobiological events throughout Ukraine, particularly on March 4 in Kyiv, Bucha, and 16 reports within Chernobyl. The risk of a nuclear accident will remain a pressing matter as the conflict continues in Ukraine. While Chernobyl has been returned to the Ukrainian government, the Zaporizhzhia plant, where spent fuel assemblies can be damaged, is still under the control of Russia [23,24]. An accident involving spent fuel assemblies could be equivalent in magnitude to the initial Chernobyl event in 1986 and requires the site to undergo constant preventive activities and monitoring. Additionally, normal preventive activities and radiation monitoring sensors have been nonfunctional during parts of the occupation, specifically in Chernobyl, and do not allow for real-time data to be received at this time [25]. OSINT reports can support governmental classified intelligence sources, gather information where formal surveillance might not be as robust or be hindered during the conflict, and provide this information in real time, which can inform timely government responses to the data presented. The significance of OSINT during the invasion, where formal information is scarce, will be to supplement more formal data sources, provide essential early warning of radiobiological events, and ensure timely emergency and public health responses. Both Epitweetr and EPIWATCH can be rapidly adapted to evolving biosecurity or other acute threats. In addition, EPIWATCH continues with the search terminology presented in this study, which routinely monitors potential radiobiological events. These systems can be used as collaborative tools with many stakeholders as a means of surveillance, both in peacetime and in active war zones.

## Appendix

Multimedia Appendix 1 Terms and associated definitions used in the search for radiobiological events in Ukraine by subtopics.

## Abbreviations

AI: artificial intelligence
ARS: acute radiation syndrome
EARS: Early Aberration Reporting System
ML: machine learning
NER: named entity recognition
NLP: natural language processing
RAD: radiation absorbed dose
REM: roentgen equivalent man
OSINT: open-source intelligence
STROBE: Strengthening the Reporting of Observational Studies
