# Supplementary material for: Open-Source Intelligence for Detection of Radiological Events and Syndromes Following the Invasion of Ukraine in 2022: Observational Study
Source: JMIR Infodemiology. 2023 Jun 28;3:e39895. doi: 10.2196/39895 (PMC10365590; doi:10.2196/39895)
Supplement: Multimedia Appendix 1 [file infodemiology_v3i1e39895_app1.docx]

**Multimedia Appendix 1. Terms and associated definitions used in the search for radiobiological events in Ukraine by subtopics.**

Data was collected from a series of search terms which are indicative of a potential radiobiological event. The search terms for individual terms and their definitions are in Table S1.

**Table S1:**Terms and associated definitions used in the search for radiobiological events in Ukraine by subtopics.

|  | English | Ukrainian | RUSSIAN | Definition |
| --- | --- | --- | --- | --- |
| Event Based Terms | Radiation | Радіація | Радиация | - |
|  | Radiological | Радіологічний | Радиологический | - |
|  | Reactor | реактор | Реактор | - |
|  | Alpha radiation | Альфа-випромінювання | Альфа-излучение | A type of ionized radiation and is not very penetrative but can cause damage to living tissue through inhalation or direct contact with exposed surface [1]. |
|  | Beta radiation | Бета-випромінювання | Бета-излучение | An additional type of ionized radiation that is more penetrative than alpha radiation but lacks the severity of alpha radiation but can cause long standing health effects [1]. |
|  | Gamma radiation | Гамма-випромінювання | Гамма-излучение | An additional type of ionized radiation that is easily the most penetrative and damaging to health [1]. |
|  | Isotope | Ізотоп | Изотоп | - |
|  | Gieger | Гігер | Гигер | Represents the Geiger-Müller tube which detects ionizing radiation [2]. |
|  | Curie | Кюрі | Кюри | The amount of ionizing radiation released energy is emitted through radiative decay [3]. |
|  | Bequerel | Бекерель | Беккерель | Radioactivity measured as one nucleus decays per second [4]. |
|  | Sievert | Зіверт | Зиверт | Ionized radiation dose measurement on the human body[5]. |
|  | REM | REM | REM | Roentgen equivalent man (rem) is a measurement for the equivalent dose in which ionized radiation effects the body [6]. |
|  | RAD | RAD | RAD | Radiation absorbed dose is the unit of measurement absorbed on object or human [7]. |
|  | Cherenkov | Черенков | Черенков | [8] |
| RADIOLOGICAL SUBSTANCE BASED TERMS | Iodine | йод | йод |  |
|  | I-131 | І-131 | І-131 | Important radioisotope which has been found in products from nuclear fission [9]. |
|  | Cesium | цезій | Цезий | Caesium isotopes can be produced through nuclear fission and can lead to radiation poisoning when exposure to the body [10]. |
|  | Cs-137 | Cs-137 | Cs-137 | Important radioisotope which has been found in products from nuclear fission and extracted from nuclear reactor waste [11]. Additionally, Cs-137 emits gamma radiation. |
|  | Cs-134 | Cs-134 | Cs-134 |  |
|  | Plutonium | плутоній | Плутоний | Is created from uranium nuclear reactors and emits alpha particles[12]. |
|  | Strontium | стронцій | Стронций | Produced through nuclear fission and linked harmful health effects[13]. |
|  | Sr-90 | Sr-90 | Sr-90 | Important radioisotope which has been found in products from nuclear fission [13] |
|  | Americium | америцій | Америций | Man-made radioactive material and when exposed to humans can concentrate in bone leading to bone marrow suppression/failure[14]. |
|  | Am-241 | Ам-241 | Ам-241 | Important radioisotope which has been found in products from nuclear fission [15] |
|  | Uranium | уран | Уран | - |
|  | Nuclear fuel | Ядерне паливо | Ядерное топливо | - |
|  | Nuclear waste | Ядерні відходи | Ядерные отходы | - |
|  | Graphite | Графіт | Графит | Graphite is commonly used in construction of modern nuclear reactors. |
| MEDICAL TERMS | Beta burn | Бета-запис / Бета опік | Бета ожог | Similar looks to sunburn, beta burn is shallow but results in large red patches on the skin [16]. |
|  | Desquamation | Десквамація | Десквамация (latin) / Шелушение (russian word) | Damage to the upper layer of skin found with acute radiation poisoning and other radiation exposures [17] |
|  | Hair loss | Втрата волосся | Выпадение волос | Common symptom for acute radiation poisoning [18] |
|  | Mucositis | Мукозит | Мукозит | A complication from chemotherapy or radiation exposure which the lining of the digestive system becomes inflamed[19]. |
|  | Gastrointestinal syndrome | Шлунково-кишковий синдром | Желудочно-кишечный синдром | Radiation-induced gastrointestinal syndrome can cause destructive and permanent damages[20] |
|  | Cardiovascular syndrome | Серцево-судинний синдром | Сердечно-сосудистый синдром | Radiation has been found to damage all parts of the heart system including the myocardium, pericardium, coronary arteries, heart valves, and the electrical system[21]. |
|  | Neurological syndrome | Неврологічний синдром | Неврологический синдром | Radiation exposure has been found to link to multiple exacerbated neurocognitive issues, in addition to, early onset migraines with acute radiation poisoning [22]. |
|  | Melena | Мелена | Мелена | A bleed in the upper gastrointestinal system. Radiation-induced gastrointestinal syndrome has been linked to cause melena. [23] |
|  | Vomiting | Блювота | Рвота | Common early onset symptom for acute radiation poisoning [18] |
|  | Lymphopaenia | Лімфопенія | Лимфопения | Blood does not contain enough white blood cells often found immunocompromised patients. Radiation exposure is found to deplete lymphocytes within the blood stream [24]. |
|  | Bone marrow suppression | Пригнічення кісткового мозку | Подавление костного мозга | Radiation exposure has been linked to bone marrow suppression with prolonged exposure linked to bone marrow failure and death [25]. |
|  | Bone marrow transplant | Пересадка кісткового мозку | Пересадка костного мозга | Increased bone marrow transplants in a given subnational location might be an indication of radiation exposure in a population. |
|  | Sepsis | Сепсис | Сепсис | With many radiation-induced immunocompromising disorders, the body is more likely to be exposed to infections. Any infection in an immunocompromised individual can lead to higher risks of sepsis. [26]. |
| RADIATION PREPAREDNESS TERMS | Potassium Iodide | Калій йодид / Йодистий Калій | Йодистый калий | Potassium iodide is commonly used when the potential for radiation exposure is high to protect the thyroid[27]. |
|  | Heavy Metal Chelation | Хелатування важких металів | Хелотирование тяжелых металлов | Used to reduce the toxic effects of metals such as ionised radiation [28]. |
|  | Calcium DTPA | Кальцій DTPA | Кальцый DTPA | Binds to radioactive materials and are then passes through the body via urine. |
|  | Zinc DTPA | Цинк DTPA | Цынк DTPA | Binds to radioactive materials and are then passes through the body via urine[29]. |
|  | Decontamination | Дезактивація/ знезараження | Деконтаминация / Обеззараживание | - |
|  | Prussian Blue | Прусський блакитний/ берлінска блакитність | Берлинская лазурь | A pill used to remove radioactive thallium and caesium from an individual[30]. |
|  | Granulocyte Monocyte Colony Stimulating Factor | Фактор, що стимулює колонію гранулоцитів моноцитів | Фактор стимулирующий колонию гранулоцитов моноцитовю / Гранулоциты Моноциты Колониестимулирующий фактор | An early treatment for radiation exposure and used in combination with other drugs to aid in improve radiation-induced bone marrow suppression [31]. |
|  | Granulocyte Colony Stimulating Factor | Фактор, стимулюючий колонію гранулоцитів | Фактор стимулирующий колонию гранулоцитов / Гранулоцитарный колониестимулирующий фактор | An early treatment for radiation exposure and used in combination with other drugs to aid in improve radiation-induced bone marrow suppression [31]. |

1. Campbell, G.S. and J.M. Norman, *Radiation basics*, in *An introduction to environmental biophysics*. 1998, Springer. p. 147-165.

2. Korff, S.A., *Geiger counters*, in *Nuclear Instrumentation II/Instrumentelle Hilfsmittel der Kernphysik II*. 1958, Springer. p. 52-85.

3. U.S.NRC. *Curie (Ci)*. 2021.

4. U.S.NRC. *Becquerel (Bq)*. 2021; Available from: <https://www.nrc.gov/reading-rm/basic-ref/glossary/becquerel-bq.html>.

5. Britannica. *Sievert*. [cited 2022; Available from: <https://www.britannica.com/technology/sievert>.

6. Britannica. *Rem*. unit of measurement [cited 2022; Available from: <https://www.britannica.com/science/rem-unit-of-measurement>.

7. U.S.NRC. *Rad (radiation absorbed dose)*. 2021; Available from: <https://www.nrc.gov/reading-rm/basic-ref/glossary/rad-radiation-absorbed-dose.html>.

8. Bolotovskii, B.M., *Vavilov–Cherenkov radiation: its discovery and application.* Physics-Uspekhi, 2009. **52**(11): p. 1099.

9. CDC. *Radioisotope Brief: Iodine-131 (I-131)*. 2022; Available from: <https://www.cdc.gov/nceh/radiation/emergencies/isotopes/iodine.htm>.

10. Williams, M., *Toxicological profile for cesium.* 2004.

11. CDC. *Radioisotope Brief: Cesium-137 (Cs-137)*. 2022; Available from: <https://www.cdc.gov/nceh/radiation/emergencies/isotopes/cesium.htm>.

12. CDC. *Radioisotope Brief: Plutonium*. 2022; Available from: <https://www.cdc.gov/nceh/radiation/emergencies/isotopes/plutonium.htm>.

13. CDC. *Radioisotope Brief: Strontium-90*. 2022; Available from: <https://www.cdc.gov/nceh/radiation/emergencies/isotopes/strontium.htm>.

14. ATSDR. *ToxFAQs™ for for Americium*. 2021; Available from: <https://wwwn.cdc.gov/TSP/ToxFAQs/ToxFAQsDetails.aspx?faqid=810&toxid=158#:~:text=Inside%20your%20body%2C%20americium%20is,the%20formation%20of%20bone%20cancers>.

15. CDC. *Radioisotope Brief: Americium-241 (Am-241)*. 2022; Available from: <https://www.cdc.gov/nceh/radiation/emergencies/isotopes/americium.htm>.

16. Modesto, A., et al., *[Evaluation and management of acute radiation dermatitis].* Cancer Radiother, 2012. **16**(5-6): p. 456-61.

17. Milstone, L.M., *Epidermal desquamation.* Journal of dermatological science, 2004. **36**(3): p. 131-140.

18. CDC. *Acute Radiation Syndrome (ARS): A Fact Sheet for the Public*. 2018; Available from: <https://www.cdc.gov/nceh/radiation/emergencies/ars.htm#:~:text=These%20symptoms%20include%20loss%20of,also%20can%20have%20skin%20damage>.

19. National Institute of Health National Cancer Institute. *NCI dictionaries: mucositis*. Available from: <https://www.cancer.gov/publications/dictionaries/cancer-terms/def/mucositis>.

20. CDC, *Acute radiation syndrome: a fact sheet for clinicians.* Center for Preparedness and Response (CPR): Georiga, 2017a [cited 16 November 2017]. Available from: <https://em> ergen cy. cd c. gov/radi ation/arsp hysic ianfa ctshe et. as p, 2017.

21. Clevland Clinic. *Radiation Heart Disease: Overview*. 2019; Available from: <https://my.clevelandclinic.org/health/diseases/17409-radiation-heart-disease-overview#:~:text=Radiation%20can%20injure%20the%20pericardium,the%20tissue%20surrounding%20the%20heart>).

22. Cross, N.E. and M.J. Glantz, *Neurologic complications of radiation therapy.* Neurol Clin, 2003. **21**(1): p. 249-77.

23. Zhang, L., et al., *Treatment of radiation-induced hemorrhagic gastritis with prednisolone: a case report.* World journal of gastroenterology, 2012. **18**(48): p. 7402-7404.

24. Cesaire, M., et al., *[Mechanisms of radiation-induced lymphopenia and therapeutic impact].* Bull Cancer, 2020. **107**(7-8): p. 813-822.

25. Green, D.E. and C.T. Rubin, *Consequences of irradiation on bone and marrow phenotypes, and its relation to disruption of hematopoietic precursors.* Bone, 2014. **63**: p. 87-94.

26. CDC. *CANCER, INFECTION AND SEPSIS FACT SHEET*. 2021; Available from: <https://www.cdc.gov/sepsis/pdfs/cancer-infection-and-sepsis-fact-sheet.pdf>.

27. CDC, *Potassium Iodide (KI).* 2021.

28. Flora, S.J.S. and V. Pachauri, *Chelation in metal intoxication.* International journal of environmental research and public health, 2010. **7**(7): p. 2745-2788.

29. CDC. *DTPA (Diethylenetriamine pentaacetate)* 2021; Available from: <https://www.cdc.gov/nceh/radiation/emergencies/dtpa.htm#:~:text=Back%20to%20Top-,How%20does%20DTPA%20work%3F,the%20body%20in%20the%20urine>.

30. CDC. *Prussian Blue*. 2021; Available from: <https://www.cdc.gov/nceh/radiation/emergencies/prussianblue.htm>.

31. Hofer, M., et al., *Granulocyte colony-stimulating factor in the treatment of acute radiation syndrome: a concise review.* Molecules (Basel, Switzerland), 2014. **19**(4): p. 4770-4778.
